# Bullous Pemphigoid Induced by Cefixime: A Rare Side Effect

**DOI:** 10.7759/cureus.74246

**Published:** 2024-11-22

**Authors:** Nicolas Sandakly, Georgio El Koubayati, Hussein Nassereddine, Fady Haddad

**Affiliations:** 1 Internal Medicine, Lebanese University Faculty of Medicine, Beirut, LBN; 2 Clinical Immunology, Lebanese University Faculty of Medicine, Beirut, LBN; 3 Pathology, Hôtel Dieu de France, Université Saint-Joseph, Beirut, LBN; 4 Internal Medicine and Clinical Immunology, Lebanese Hospital Geitaoui - University Medical Center, Beirut, LBN

**Keywords:** autoimmune bullous disorders, case report, cefixime, drug-induced bullous pemphigoid, drug reaction

## Abstract

Bullous pemphigoid (BP) is the most prevalent autoimmune subepidermal blistering disease of the skin and mucous membranes. This disease typically affects the elderly and manifests with pruritus and localized or, most commonly, generalized bullous lesions. Numerous studies have established the association between BP and oral antidiabetic agents, particularly dipeptidyl peptidase 4 (DPP4) inhibitors, diuretics, and certain antibiotics, notably levofloxacin and cephalexin. In this report, we present a case of an 85-year-old female who presented with diffuse vesicles and bullae on her trunk and extremities five days after completing a four-week course of cefixime for pyelonephritis. Clinical examination and histopathological analysis confirmed the diagnosis of BP. The patient responded well to topical and systemic corticosteroids. This article presents the first documented case of BP induced by cefixime and underscores the importance of considering medication-induced BP in elderly patients presenting with blistering eruptions.

## Introduction

Bullous pemphigoid (BP) is an autoimmune subepidermal blistering disease predominantly observed within the geriatric population, although occurrences among children and younger adults have also been reported. It is caused by IgG autoantibodies, occasionally IgA, IgM, and IgE, targeting components of the adhesion complex within the basement membrane zone (BMZ), resulting in subepidermal blistering. The annual incidence of BP ranges from six to 43 cases per million [1]. The diagnosis is confirmed by immunofluorescence of a skin biopsy procured approximately 1 cm from a fresh blister. Management strategies for BP include topical and systemic therapy to control pruritus and skin eruptions and prevent the risk of recurrence, given the substantial morbidity and mortality associated with the condition. Various studies have identified risk factors for BP, including cerebrovascular diseases, Parkinson’s disease, epilepsy, and multiple sclerosis [1,2]. Recent meta-analyses suggest certain drugs as potential triggers for BP, notably dipeptidyl peptidase 4 (DPP4) inhibitors and immune checkpoint inhibitors [3,4]. However, uncertainty remains regarding the role of other drugs, such as antibiotics and anti-inflammatories, on BP onset. In this article, we present a case wherein BP was triggered by the intake of cefixime, a third-generation cephalosporin, prescribed for a urinary tract infection. This case represents the first documented instance of BP attributed to cefixime ingestion.

## Case presentation

**Figure 1:**
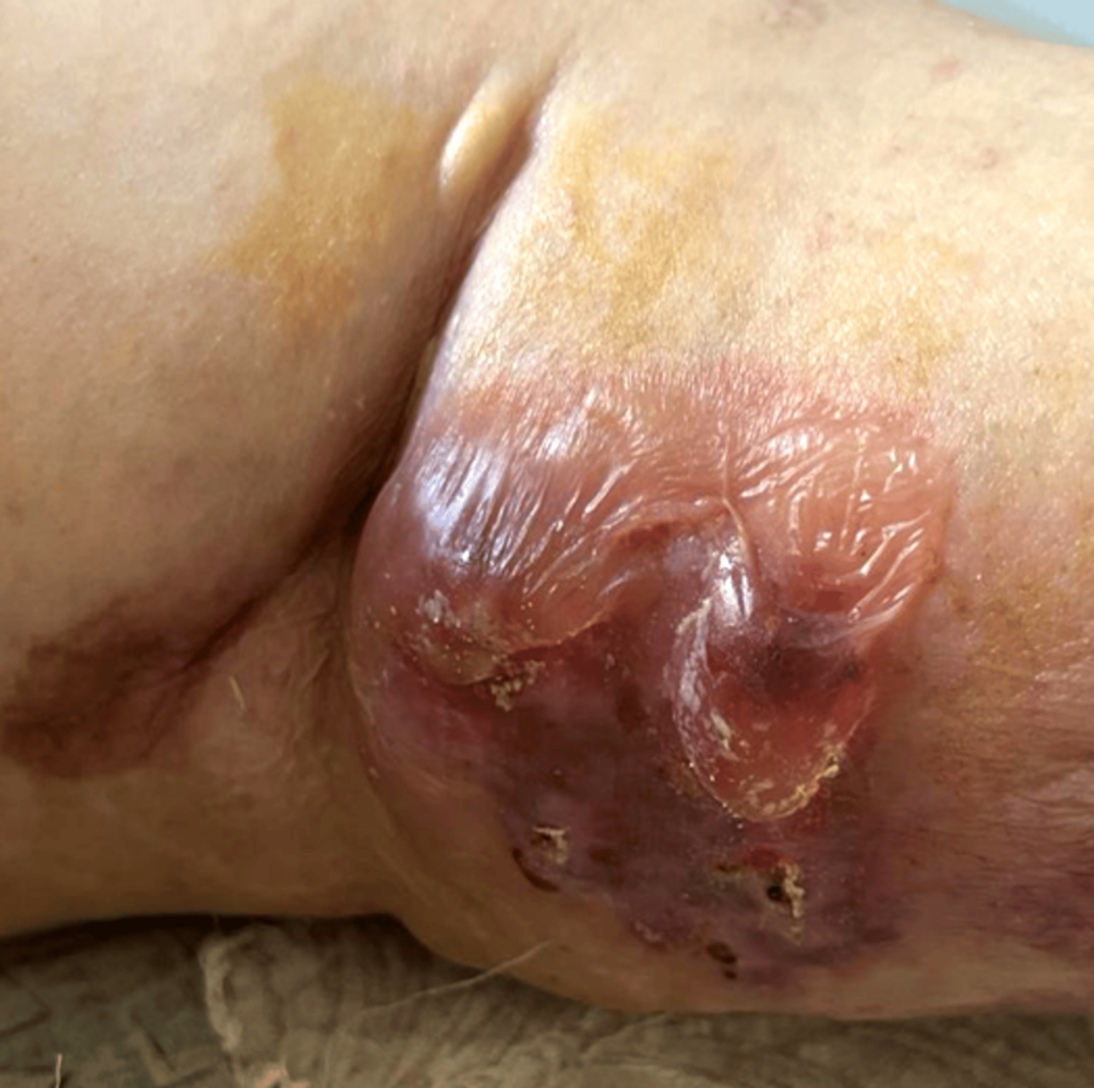
Tense fluid-filled bullae on the anterior aspect of the right leg.

**Figure 2:**
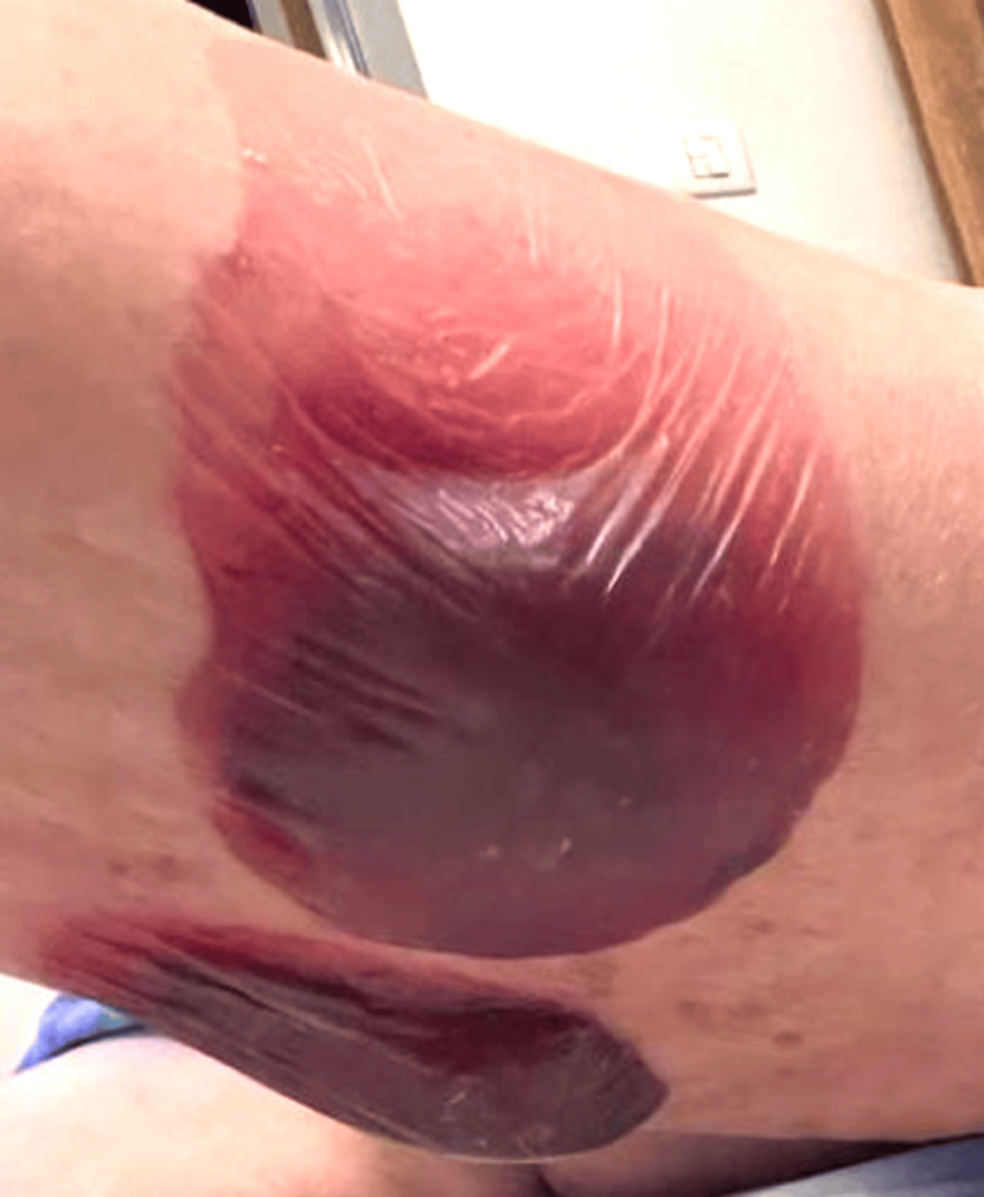
Hemorrhagic tense bullae on the left forearm.

**Figure 3:**
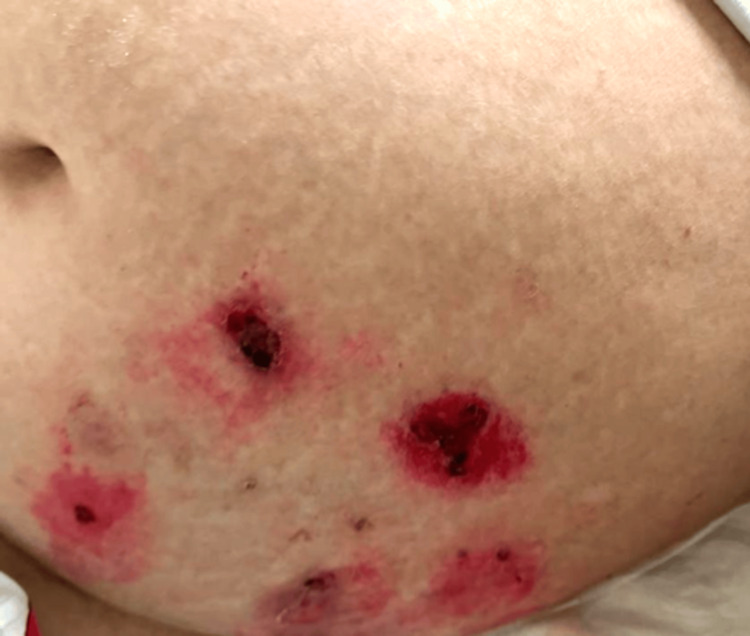
Erosions on the abdomen.

An 85-year-old female patient presented to the Lebanese Hospital Geitaoui - University Medical Center (LHG-UMC) with a medical history significant for hypertension, dyslipidemia, and atrial fibrillation for a two-week history of diffuse vesicles and bullae on her trunk and extremities. The vesicles appeared five days after finishing a course of 28 days of cefixime, a third-generation cephalosporin that was prescribed for pyelonephritis diagnosed a month prior. Upon presentation, the patient was afebrile, and vital signs were stable. Skin examination revealed diffuse erythematous crusted plaques, serous fluid-filled bullae, and hemorrhagic blisters with ruptured ones over the trunks and the upper and lower extremities (Figures 1-3).

**Figure 4:**
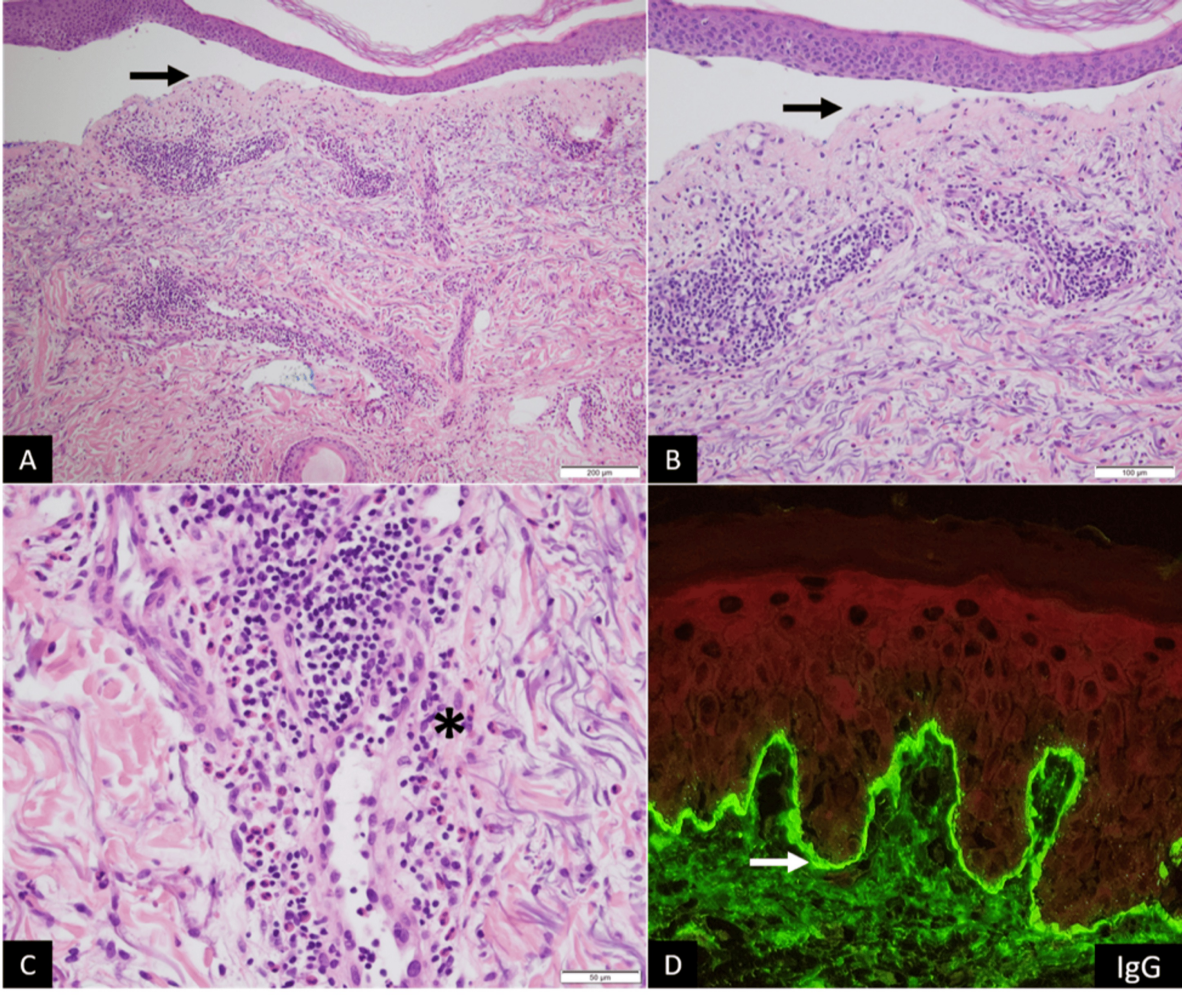
Pathology results of the skin biopsy. Microscopic features showing (A, B) a sub-epidermal blister (black arrow) and a moderately dense superficial perivascular and interstitial inflammatory infiltrate (hematoxylin and eosin staining, ×100 (A) and ×200 (B)). (C) Moderately dense perivascular and interstitial lymphocytic infiltrate with numerous eosinophils (asterisk) (hematoxylin and eosin staining, ×400). (D) Direct immunofluorescence studies revealed positive immunostaining with IgG in a linear pattern along the dermoepidermal junction (white arrow).

The lesions were itchy and painful. Nikolsky’s sign was negative. Mucous membranes were not affected. Complete blood count was normal. No eosinophilia was present. Liver and renal function tests were normal. C-reactive protein (CRP) was 15 mg/L (reference range: <6 mg/L) and erythrocyte sedimentation rate (ESR) was prolonged at 65 mm/1st hour. A skin biopsy was performed (Figure 4) that revealed a subepidermal blister. In the dermis, there was a moderately dense superficial perivascular and interstitial lymphocytic infiltrate with numerous eosinophils and scattered neutrophils. Direct immunofluorescence studies revealed positive immunostaining with C3 and IgG in a linear pattern along the dermoepidermal junction.

Culture taken from the skin lesion upon admission yielded negative findings. Taken together, the clinical presentation and the histopathological findings, were consistent with the diagnosis of BP. Serology for human immunodeficiency virus (HIV), hepatitis B virus (HBV), hepatitis C virus (HCV), and cytomegalovirus (CMV) yielded negative results. The antinuclear antibody (ANA) test came back negative. A contrast-enhanced computed tomography (CT) of the chest, abdomen, and pelvis was performed to rule out paraneoplastic pemphigus, which returned negative findings. According to Naranjo’s adverse drug reaction algorithm (Table 1), a score of 6 was obtained.

**Table 1:** Naranjo’s score of 6 in our patient indicating probable bullous pemphigoid. Naranjo’s score: ≥9 = definite; 5 to 8 = probable; 1 to 4 = possible; ≤0: doubtful.

Question	Yes	No	Do not know	Results
1. Are there previous conclusive reports on this reaction?	+1	0	0	No
2. Did the adverse event appear after the suspected drug was administered?	+2	-1	0	Yes
3. Did the adverse event improve when the drug was discontinued or a specific antagonist was administered?	+1	0	0	Yes
4. Did the adverse event reappear when the drug was readministered?	+2	-1	0	Do not know
5. Are there alternative causes that could on their own have caused the reaction?	-1	+2	0	No
6. Did the reaction reappear when a placebo was given?	-1	+1	0	Do not Know
7. Was the drug detected in blood or other fluids in concentrations known to be toxic?	+1	0	0	Do not know
8. Was the reaction more severe when the dose was increased or less severe when the dose was decreased?	+1	0	0	Yes
9. Did the patient have a similar reaction to the same or similar drugs in any previous exposure?	+1	0	0	No
10. Was the adverse event confirmed by any objective evidence?	+1	0	0	No
Total score				6

**Figure 5:**
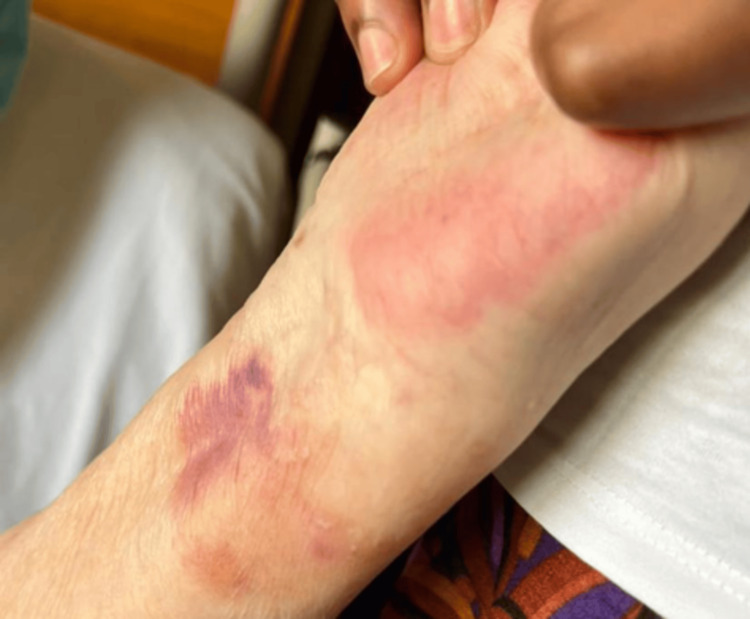
Healed blisters on the forearm.

BP probably caused by cefixime was considered, further supported by the fact that no other medication had been introduced in the past three months. A review of her medication did not reveal any drug that could be associated with BP, such as DDP-4 inhibitors or furosemide. Additionally, no over-the-counter medications or herbal products had been ingested recently. The patient was started on topical potent steroids (clobetasol dipropionate 15 g applied twice daily on affected areas) and after four days of treatment, the cutaneous lesions improved. However, three days later, a new itching bullous eruption developed on the trunk, so methylprednisolone 40 mg twice daily was added to the topical cream. Over the next few days, cutaneous lesions improved, and, in some places, the blister fluid was absorbed and the skin surface shrunk (Figure 5).

The patient was discharged home on prednisolone 30 mg once a day and tapered down slowly over the next six months in combination with azathioprine 50 mg twice a day. After four months, no new blister formation was found.

## Discussion

There is a large number of medications reported to induce BP since the first documented case of drug-induced BP back in 1970, involving an 11-year-old boy who received sulfasalazine. In a systematic review of drug-associated BP [5], the strongest evidence was observed with DPP4 inhibitors, an oral hypoglycemic agent used to treat type 2 diabetes mellitus. The first Finnish nationwide registry study published in 2018 [6] reported a 10-fold increased risk of BP associated with vildagliptin, with a mean latency from exposure to diagnosis of approximately 449 days. BP is more prevalent in patients receiving anti-programmed cell death-1 (anti-PD1) therapy compared to the general population. In a systematic review encompassing all cases of bullous disorders associated with anti-PD1 therapy, of the 29 reported cases, BP was described in 15 cases [7]. Antibiotics are among the first class of medications associated with BP. Numerous case reports in the literature have directly linked levofloxacin [8], ciprofloxacin [9], amoxicillin [10], and first-generation cephalosporin cephalexin [11] to BP. To the best of our knowledge, there has been no previously reported case of cefixime-induced BP in the literature. A comprehensive search of the literature on the PubMed database using the keywords pemphigoid, bullous pemphigoid, and drug-induced bullous pemphigoid yielded over 300 reported cases implicating various classes of drugs in BP onset, with no documented association with third-generation cephalosporin, in particular, cefixime. Herein, we describe the first case of an 85-year-old female patient with blistering eruption with findings consistent with a diagnosis of BP occurring after recent cefixime ingestion. While our patient was not re-challenged with the drug in question due to ethical considerations, the rapid response to treatment and the absence of relapse further support this association. Various theories have been proposed regarding the mechanism by which drugs can induce BP. Sulfur-containing drugs, exemplified by penicillamine and captopril, can directly cause acantholytic splitting leading to the disruption of the dermoepidermal junction without any immune-mediated process by interacting between the sulfhydryl groups in desmosomes and BMZ [12]. Another possible theory considers that certain drugs may alter the antigenicity of structures within the lamina lucida, acting as a hapten that binds to and modifies protein molecules in the basement membrane, thereby exposing hidden antigenic sites [13]. A retrospective study conducted by Patsatsi et al. [14] reported statistically significant increased titers of circulating anti-BP180 auto-antibodies in BP patients receiving various systemic medications before disease onset when compared to BP patients who never received any medications, supporting the hypothesis of drug-induced antigenic alterations acting as hapten. Inhibition of regulatory T cells by immune checkpoint inhibitors that lead to the proliferation of humoral cells and the hyperproduction of auto-antibodies against the BP antigens is another proposed theory for the pathogenesis of drug-induced BP [15]. Since cefixime does not contain sulfhydryl or phenol group, we postulate that cefixime may have acted as hapten in our patient. Our patient was advised not to use this drug for any future indications and follow-up visits revealed remission of the lesions.

## Conclusions

This case adds to the growing body of literature documenting various medications associated with BP onset. We have presented the first case of cefixime-induced BP in a patient who developed bullous lesions shortly after ingesting cefixime with complete resolution of the lesions and the absence of relapse during follow-up. This case highlights the importance for clinicians to consider drug-induced BP in patients presenting with blistering eruptions, especially after recent medication exposure, and to consider cefixime as a potential offending drug in BP cases. Further studies are needed to validate our observation and elucidate the underlying pathogenesis.
